# Engineered zero-order drug release from degradable PEG hydrogels – A rapamycin case study

**DOI:** 10.1177/08853282251410673

**Published:** 2026-01-06

**Authors:** Lage Ahrenstedt, Anel Oosthuysen, Peter Zilla, Jaco Theron, Deon Bezuidenhout

**Affiliations:** 1 Cardiovascular Research Unit, 37716 University of Cape Town, Cape Town, South Africa

**Keywords:** hydrogels, PEGylation, rapamycin, drug delivery, zero-order elution, release kinetics

## Abstract

This study describes the derivatization of Rapamycin (Ra) with acryloyl chloride (AcCl) and iodoacetic acid (IAA), yielding hydrolysis-susceptible esters designed for controlled drug release at physiological pH. These esters were further conjugated to thiolated polyethylene glycols (PEGs), yielding compounds with enhanced water solubility, pendant thiol groups and with variation in the number of methylene groups between the ester and thioether moieties. Hydrogels were subsequently formed via conjugate addition reactions using multi-arm PEG macromers, specifically 8-arm PEG acrylates or vinyl sulphones, alongside thiolated PEG crosslinkers. The primary focus was to elucidate the impact of structural modifications surrounding the thioether ester linker on drug release kinetics. In vitro release studies demonstrated zero-order Ra elution over 7–19 days, modulated by gel architecture. Notably, Ra incorporated via α-thioether ester bonds exhibited significantly faster release than their β-thioether ester counterparts, with release rate increases of 11% and 31%, respectively, across the gel assemblies examined. This behavior was attributed to the electron-withdrawing effect of the adjacent thioether group, which enhanced ester hydrolysis. Additionally, creating a hydrogel more prone to swelling and degradation (by using the PEG acrylate multi-arm instead of the PEG vinyl sulphone equivalent) increased the overall drug release rate due to higher water uptake within the gel matrix. An alternative strategy involved Ra-based crosslinking, where Ra, di-functionalized with IAA, acted as a crosslinker for the PEG thiol multi-arm molecules. This assembly exhibited a biphasic release profile, initially mimicking the linear zero-order release of Ra mono-iodoacetic ester crosslinked with PEG acrylates, followed by an exponential burst phase. These findings provide critical insights into hydrogel design strategies for tailoring drug release kinetics, paving the way for advanced controlled drug delivery applications.

## Introduction

Rapamycin (Ra), also known as Sirolimus, is a bacteria-derived macrolide compound widely recognized for its antifungal properties and potent immunosuppressive activity,^1^ as well as anti-inflammatory effects.^2^ Its primary mechanism of action involves the inhibition of the mammalian target of rapamycin (mTOR), leading to mitigation of cardiovascular disease progression.^3^ Ra has been shown to alleviate vascular inflammation,^2^ reduce restenosis following vascular interventions by limiting the migration and proliferation of vascular smooth muscle cells,^4^ and counteract myocardial infarction by attenuating pathological coronary hypertrophy^5^ and adverse left ventricular remodeling.^6^ Ra shows promise in the treatment of cancer,^7^ is used as immunosuppressant after organ transplant,^8^ and is also investigated as immunosuppressant in the treatment of COVID-19 and similar diseases that develop a cytokine storm.^9^

Low aqueous solubility of hydrophobic drugs such as Ra is a common challenge in drug delivery and clinical efficacy. Various strategies have been explored to enhance drug solubility, with one widely used approach being conjugation with hydrophilic polymers such as polyethylene glycol (PEG). PEGylation improves solubility, extends circulation time, and protects drugs (especially peptides and proteins) from enzymatic degradation.^10^

The mode of drug delivery plays a crucial role in determining therapeutic outcomes and the occurrence of adverse effects. For instance, systemic administration of Ra for restenosis prevention following stent implantation has been associated with minor adverse effects in approximately 20% of treated patients.^11^ Also, a retrospective study for evaluating the effectiveness of Ra as an immunosuppressant after kidney transplantation, reported that more than 10% of patients experienced side effects and the treatment was discontinued in 46% of patients due to serious proteinuria, ulcers and edema.^8^ In contrast, localized delivery through Ra-eluting stents has demonstrated improved safety, with no serious adverse effects reported at 12-months follow-up of patients treated for vertebral artery stenosis.^12^ This underscores the advantages of site-specific drug release platforms, including in situ formed hydrogel-based delivery systems.

The ability of hydrogels to encapsulate and gradually release therapeutic agents has made them valuable in targeted drug delivery applications. Hydrogels are widely applied in the biomedical field due to their tunable properties, which allows for precise control over mechanical strength, degradation behavior and bioactivity, making them highly suitable as sustained drug delivery platforms for applications such as wound dressings and implant coatings.^13,14^

Hydrogels with ester bond crosslinks primarily degrade through hydrolysis of these linkages. While esters are relatively stable under neutral pH conditions,^15^ their degradation rate can be modulated by specific chemical environments. Notably, incorporating adjacent thioether groups has been found to destabilize ester bonds, leading to accelerated hydrolysis^16,17^ caused by electron-withdrawing effects.^18^ Schoenmakers et al. noted that introducing methylene groups between the carbonyl and thioether also impacts on the degradation rate.^18^ Additional destabilization can be induced by integrating electron-withdrawing functional groups, such as specific amino acid residues, in line with the thioether.^19^ Degradation kinetics are also influenced by crosslink density, with higher densities correlating with slower degradation.^20^

Drugs can be incorporated in a hydrogel by either blending the free drug into the polymeric matrix, e.g. Ra-loaded polymer matrices used as stent coatings,^21–23^ or by covalently coupling the drug to the gel matrix, e.g. chemotherapeutic^24^ and anti-inflammatory drugs^25^ released from hydrogels. Entrapped drugs typically follow a biphasic release profile - an initial diffusion-driven phase followed by a degradation-induced release phase.^21–23^ However, Ahrenstedt et al. showed that zero-order release kinetics can be achieved by covalently attaching the drug to the polymer matrix, ensuring a constant, steady release rate.^26^ Zero-order drug release presents multiple therapeutic advantages, including maintaining steady-state drug levels, reducing toxicity risks, and improving overall treatment efficacy. Maintaining a stable drug concentration and ensuring prolonged therapeutic activity is essential for optimizing patient outcomes.^27^

Benjamin et al. chemically modified Ra to produce rapalogues suitable for oral and intravenous administration.^28^ Conjugation of Ra to small molecules^29^ and linear PEG macromolecules^30^ was also found to enhance solubility and cellular uptake of Ra. However, no reports could be found describing covalent incorporation of Ra in a hydrogel matrix, as is widely described for numerous other drugs.^20,25,26,31^

PEG is regarded as biologically inert and undergoes only minimal enzymatic degradation in vivo. PEG is mainly converted to small metabolites, such as mono- and dicarboxylic acids and hydroxy acids, as a result of hydrolytic cleavage. While trace amounts of these oxidation products have been detected in plasma and urine, the majority of PEG is excreted unchanged. Low-molecular-weight PEG (<20 kDa, such as used in this study) is predominantly eliminated via renal excretion, whereas larger PEGs (>30 kDa) are more likely to follow the biliary elimination route.^32^ Very large PEGs (>50 kDa) have been reported to accumulate in body tissues.^33^

This study explores methods for achieving controlled and prolonged Ra release by covalently integrating it into PEG-based hydrogels, following methodologies established by our research group.^26,31^ Three distinct hydrogel formulations are investigated, each employing different crosslinking strategies to fine-tune drug release kinetics.

Ultimately, these hydrogel systems are intended to support post-myocardial infarction recovery by enabling direct myocardial injection, a therapeutic strategy previously validated by our team.^34,35^ Additionally, these hydrogels also hold promise for applications in vascular grafts and stent coatings, with the potential to enhance patency and reduce restenosis.

## Materials and methods

### Materials

Rapamycin (Ra) was obtained from Zhou Fang Pharm Chemical (Shanghai, China), 10PEG-4OH (10 kDa, 4 arms) and 20PEG-8OH (20 kDa, 8 arms) were obtained from Nektar Therapeutics (Huntsville, USA). General reagents and solvents (reagent grade or higher) were obtained from commercial sources.

The functional groups of the hydrogel precursors are abbreviated as follows: thiol group (SH), acrylate group (Ac), vinyl sulphone group (VS), iodoacetic ester group (IAE) and di-iodoacetic ester groups (DiIAE).

Phosphate buffered saline solution with enhanced buffering capacity (150 mM; PBS-2) was used as solvent for hydrogel preparation. Standard phosphate buffered saline (150 mM; PBS-1) was employed as medium for all swelling and drug elution experiments.

### Analytical methods

#### Statistical analysis

Statistical analyses were performed using Microsoft Excel. All data are presented as mean ±2 × standard deviation (SD) for n = 3 gels per group.

Drug release mechanisms were classified using standard kinetic models. Release was classified as zero-order when the entire release curve (cumulative release vs time) could accurately be described by a linear regression model, with the slope of the straight line representing the zero-order release rate constant (k_0_). If a linear relationship between ln (fractional drug remaining) vs time was observed, the release profile was instead classified as first-order. A coefficient of determination (R^2^) > 0.95 was considered a significant fit for either zero- or first-order models. Formulations in which the majority of Ra was released within the first few days, and whose profiles could not be adequately described by the first-order model, were classified as exhibiting burst release and were not subjected to further kinetic modelling.

Zero-order release rate constants (k_0_) were compared between assemblies differing in their linkers, using an unpaired two-tailed Student’s t-test. A *p*-value <0.05 was considered statistically significant.

#### Nuclear magnetic resonance (NMR) analysis

Spectra were recorded on a Varian spectrometer operating at 300 MHz. For the ^1^H NMR spectra, only shifts affecting terminal protons due to synthesis are reported. When necessary for further clarification, ^13^C NMR peaks are also reported. Tetramethyl silane (TMS) served as the reference peak at 0 ppm.

#### Mass spectrometry (MS) analysis

The molecular weights of modified Ra compounds ((**3**), (**4a**) and (**4b**)) were confirmed by MS on a Waters API Q-TOF Ultima mass spectrometer. The samples were dissolved in acetonitrile (MeCN), diluted with 50% MeCN in water to 20 μg/ml and introduced (2 μl) to the MS using a Waters UPLC pump at a flow rate of 0.2 ml/min in a mixture of MeCN and 0.1% formic acid (80:20). The samples were ionized by electro spray ionization^36^ in positive mode (capillary voltage: 3.5 kV, cone voltage: 35, RF1: 40, source temperature: 100°C, desolvation temperature: 350°C, desolvation gas flow: 350 l/h and cone gas flow: 50 l/h). Data was acquired at a mass/charge window of 300-1500 and processed with the “Masslynx 4.1” software.

The amount of mono-, di- and tri-substitution of compound (**3**) was quantified by separating the crude reaction mixtures on an Xbridge C18 column (dimensions: 50 × 2.1 mm, Waters) in line with the ESI-MS system. The substituted compounds were washed out from the column by an increasing gradient of MeCN in 0.1% formic acid (20-100% in 12 min, 0.35 ml/min), quantified by UV absorption at 280 nm and assigned by ESI LC-MS.

#### High pressure liquid chromatography (HPLC)

Modified Ra compounds ((**3**), (**4a**) and (**4b**)) were analyzed with HPLC on an Agilent 1200 series with respect to purity. The samples were loaded (5 μl, 1 mg/ml MeOH), separated into their constituents on a Jupiter C18 column (dimensions: 250 × 4.6 mm, porosity: 300 Å, particle size: 5 μm) from Phenomenex using a linear gradient of MeCN in distilled water (0-100% in 35 min, 0.7 ml/min) and detected with a diode array detector. The data was processed with the “Chemstation for LC 3D systems” software.

### Synthesis of the gel precursors

**Figure 1.**
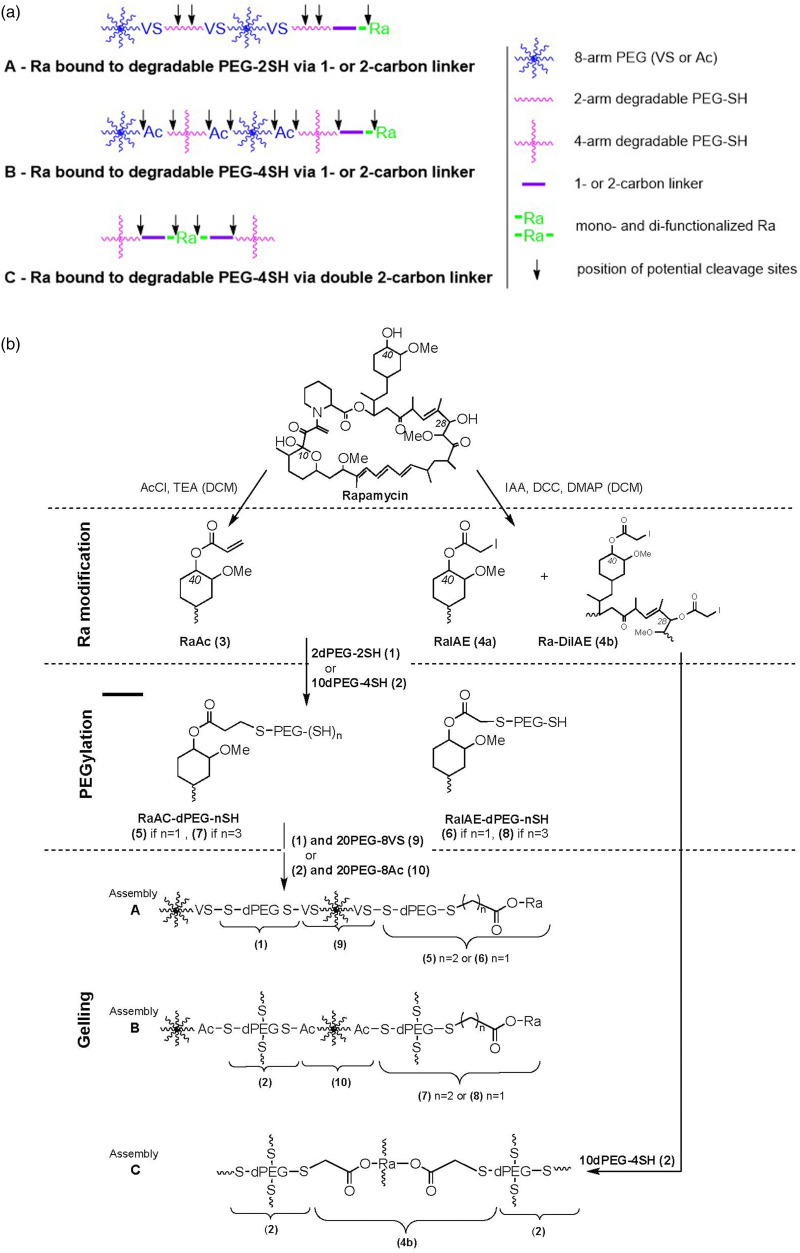
(a) Schematic diagram of the Ra-PEG gel architecture of Assemblies A, B and C; and (b) Scheme for PEGylation of Ra and gel formation from the precursors.

Figure 1(a) provides a simplified schematic diagram of the gel architecture and drug incorporation, while a detailed overview of the synthesis steps, as described below, is presented in Figure 1(b).

#### Synthesis of hydrolytically degradable dPEG-SH (2dPEG-2SH (1) and 10dPEG-4SH (2))

**Figure 2.**
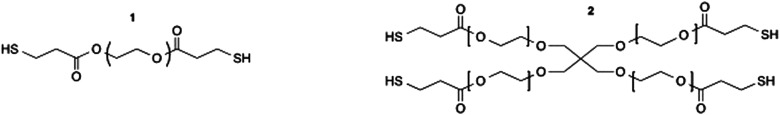
Structure of hydrolytically degradable dPEG-SH - 2dPEG-2SH (1) and 10dPEG-4SH (2).

Compounds (**1**) and (**2**) (Figure 2) were synthesized by conjugation of 2 kDa linear PEG (2PEG-2OH) and 10 kDa star-shaped 4-arm PEG (10PEG-4OH), respectively, with 3-mercaptopropionic acid, following a method adapted from Nie et al.^37^ Details of the synthesis are given in the supplementary information (S1-Synthesis of 2dPEG-2SH (1) and S2-Synthesis of 10dPEG-4SH (2)).

#### Synthesis of rapamycin acrylate (RaAc (3))

Ra (304 μmol) and triethyl amine (TEA; 610 μmol) were dissolved in dichloromethane (DCM; 20 ml) and acryloyl chloride (AcCl; 606 μmol in 10 ml DCM) was slowly added under argon atmosphere. The reaction was followed by thin layer chromatography (TLC), and additional amounts of AcCl and TEA were added (total addition 8 eq/Ra) until all Ra (R_f_ ≈ 0.3) was consumed. The crude product was loaded to a silica packed column and washed with an increasing gradient of ethyl acetate (EtOAc) in n-hexane (Hex), until the product eluted at 50%. All fractions containing the product (R_f_ ≈ 0.7) were pooled and dried. The product was further dissolved in *tert*-butyl methyl ether, filtered and precipitated in *n*-heptane to yield a slightly off-white powder.

#### Synthesis of rapamycin iodoacetic ester (RaIAE (4a)) and rapamycin di-iodoacetic ester (RaDiIAE (4b))

Ra (273 μmol), 4-dimethylamino pyridine (DMAP; 16 μmol), iodoacetic acid (IAA, 328 μmol) and N,N′-dicyclohexyl carbodiimide (DCC; 328 μmol) were dissolved in DCM (15 ml), and stirred overnight on ice. The reaction was followed by TLC and the incomplete consumption of Ra (R_f_ ≈ 0.3) led to the addition of more IAA (0.2 eq/Ra) and DCC (0.2 eq/Ra). The crude product was loaded to a silica packed column and washed with an increasing gradient of EtOAc in Hex, until the products eluted at 40%, with only a small overlap. All fractions containing product (**4a**) (R_f_ ≈ 0.7) and product (**4b**) (R_f_ ≈ 0.9) were pooled separately and dried to yield off-white powders.

#### Synthesis of RaAc-dPEG-SH (5)

Compound (**3**) (7.7 μmol) was dissolved in MeCN (200 μl) and a solution of (**1**) (17.0 mg, 7.7 μmol, in 100 μl of 0.1 M NaHCO_3_) was added. The reaction mixture was incubated at 37°C for 1 h prior to freeze-drying.

#### Synthesis of RaIAE-dPEG-SH (6)

Compound (**4a**) (8.5 μmol) was dissolved in a mixture of MeCN (200 μl) and methanol (200 μl) and a solution of (**1**) (18.5 mg, 8.4 μmol, in 200 μl of 0.1 M NaHCO_3_) was added. The reaction mixture was incubated at 37°C for 2 h prior to freeze-drying.

#### Synthesis of RaAc-dPEG-3SH (7)

Compound (**3**) (26.8 μmol) was dissolved in MeCN (1.5 ml) and a solution of (**2**) (273.0 mg in 750 μl of 0.1 M NaHCO_3_, 26.8 μmol) was added. The reaction mixture was incubated at 37°C for 1 h prior to freeze-drying. The crude product was redissolved in DCM (2.2 ml) and precipitated in cold diethyl ether (25 ml) to yield the product.

#### Synthesis of RaIAE-dPEG-3SH (8)

Compound (**4a**) (29.5 μmol) was dissolved in MeCN (1.6 ml) and diluted with of a solution of (**2**) (300.6 mg in 800 μl of 0.1 M NaHCO_3_, 29.5 μmol). The reaction mixture was incubated at 37°C for 1 h prior to freeze-drying. The crude product was redissolved in DCM (3 ml), filtered and precipitated in cold diethyl ether (25 ml) to yield the product.

The amount of Ra in the PEG conjugates (compounds (**5**), (**6**), (**7**) and (**8**)) was quantified by dissolving each respective compound in a 1:1 mixture of PBS-1 and ethanol. Subsequently, the absorbance of these solutions was measured at λ = 279 nm and the Ra concentration was determined utilizing a standard curve generated from serial dilutions of Ra.

#### Synthesis of 20PEG-8VS (9) and 20PEG-8Ac (10)

**Figure 3.**
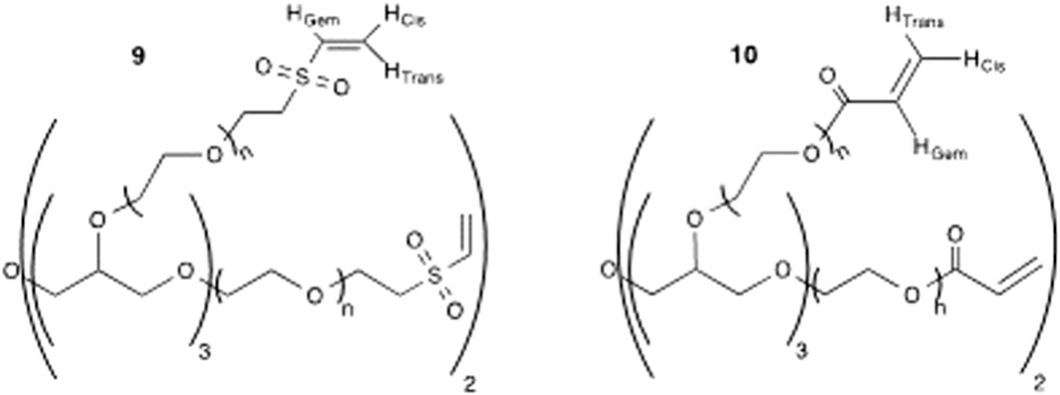
Structure of multi-arm PEG crosslinkers - 20PEG-8VS (9) and 20PEG-8AC (10).

The synthesis protocols for compound (**9**) and (**10**) (both 20 kDa, 8 arms, Figure 3) were previously described^31^ and details are given in the supplementary information (S3 - Synthesis of 20PEG-8VS (9) and S4 - Synthesis of 20PEG-8Ac (10)).

### Gel formation

Three gel assemblies were made (shown in Figure 1):

**A**: RaAc (**3**) or RaIAE (**4a**), PEGylated with 2dPEG-2SH (**1**), yielding (**5**) or (**6**), which was co-polymerized with 2dPEG-2SH (**1**) and 20PEG-8VS (**9**) → **RaAc-SH** gels or **RaIAE-SH** gels
**B:** RaAc (**3**) or RaIAE (**4a**), PEGylated with 10dPEG-4SH (**2**), yielding (**7**) or (**8**), which was co-polymerized with 10dPEG-4SH (**2**) and 20PEG-8Ac (**10**) → **RaAc-3SH** gels or **RaIAE-3SH** gels
**C**: non-PEGylated RaDiIAE (**4b**), co-polymerized with 10dPEG-4SH (**2**) → **RaDiIAE** gels

For gel assemblies A and B, three variations were prepared for each:

**Bound:** Gels with Ra covalently bound using either an Ac linker (Ac-Bound), or an IAE-linker (IAE-Bound).

**Trapped:** Gels in which equivalent amounts of unmodified Ra (instead of Ac-Bound or IAE-Bound) were physically entrapped within the gel network during crosslinking.

**Control:** Gels without Ra.

#### Assembly A gels

***(Ac-Bound and IAE-Bound):*** 20PEG-8VS (**9)** was dissolved in PBS-2 (200 mg/ml; 151 μl and 170 μl, respectively) and solutions of either (**5**) or (**6**) were added (loading density of 1 Ra per 10 VS, 102 mg/ml; 40 μl and 115 mg/ml; 40 μl, respectively)**.** The mixtures were incubated at 37°C for 20 min in closed containers prior to the addition of crosslinker (**1**) (9 SH per 10 VS, 82 mg/ml PBS-2; 131 μl and 150 μl, respectively).

**Trapped:** Unmodified Ra (1 Ra per 10 VS in 20 μl EtOH) replaced the solutions of (**5**) and (**6**) above.

**Control:** These gels were produced from gel precursors (**1**) (80 mg/ml PBS-2; 153 μl) and (**9**) (200 mg/ml PBS-2; 153 μl) in stoichiometric ratio.

Gels (n = 3 each) were formed by allowing 100 μl drops of mixed pre-gel solutions to gel for 40 min at 37°C, 100% RH, yielding a drug load of 5, 7 and 9 mg/g gel, respectively, for A1 Ac-Bound, IAE-Bound and A2 Trapped.

#### Assembly B gels

***(Ac-Bound and IAE-Bound)*:** 20PEG-8Ac (**10**) was dissolved in PBS-2 (200 mg/ml; 145 and 163 μl, respectively) and individual solutions of either (**7**) (175 mg/ml; 76 μl) or (**8**) (191 mg/ml; 93 μl), corresponding to a loading density of 1 Ra per 10 AC were added. Thereafter, the crosslinker (**2**) (300 mg/ml; 69 and 70 μl, respectively; 7 SH per 10 Ac) was added**.**

**Trapped:** Unmodified Ra solution (1 Ra per 10 Ac, 56 mg/ml EtOH; 20 μl) replaced the solutions of (**7**) and (**8**) above.

**Control:** These gels were produced from the gel precursors (**2**) (204 mg/ml; 138 μl) and (**10**) (200 mg/ml; 138 μl) in stoichiometric ratio, in PBS-2.

Gels (n = 3 each) were formed by allowing 50 μl drops of pre-gel solutions to gel for 45 min at 37°C, 100% RH, yielding a drug load of 5, 7 and 7 mg/g gel, respectively, for B1 Ac-Bound, IAE-Bound and B2 Trapped.

#### Assembly C gels

Compound (**4b**) (6.9 mg, 11.0 μmol IAE) was dissolved in MeCN (140 μl) and a solution of (**2**) (400 mg/ml 0.1 M NaHCO_3_, 70 μl, 11.0 μmol SH) was added. Gels (n = 3) were formed by allowing 50 μl drops of pre-gel solutions to gel for 18h at 37°C, 100% RH, yielding a drug load of 37 mg/g gel.

The theoretical drug content in the gels (mg Ra/g gel) was calculated using the weighed amount of drug-carrier conjugate (compound (**5**), (**6**), (**7**) or (**8**)), the molecular weight of the drug in relation to the conjugate compound and the measured volumes in each step of the mixing procedure. The calculated drug content was subsequently divided by the measured gel weight, obtained prior to submersion.

### Gel swelling and rapamycin elution

The resulting gels were weighed (W_g_) individually, immersed in PBS-1 (2 ml, pH 7,4) in sealed containers, and incubated at 37°C for evaluation of swelling and drug elution.

At regular intervals the swollen gels were removed, carefully blotted and weighed (W_s_), after which the supernatant was replaced by fresh buffer. The collected supernatant was diluted with ethanol (EtOH; 1:1), the absorbance (λ_Ra_ = 279 nm) measured and the amount of released Ra calculated using a standard curve made from serial dilutions of a stock Ra solution (PBS-1/EtOH; 1:1). Pooled supernatant from the Control gels was used as background for gel assemblies A and B; for assembly C, a mixture of PBS-1 and EtOH (1:1) was used instead. After a period of degradation, the gels became too weak to be handled manually, and the remaining drug load was released by addition of NaOH (1 ml, 1M).

## Results

### Synthesis of gel precursors

#### Ra functionalization

The functionalization of Ra with PEG proceeded through a two-step modification process (Figure 1(b)). In the first step, Ra was reacted with either AcCl or IAA to introduce functional groups that facilitate conjugation with thiolated PEGs via conjugate addition or nucleophilic substitution, respectively. The hydroxyl groups on the Ra molecule served as the primary reaction sites for these modifications. This process produced mono-, di-, and tri-substituted derivatives, which were characterized using HPLC and ESI LC-MS, results given in supplementary information (S5 – HPLC and LC-MS characterization of compounds (3), (4a) and (4b)). The mono-functionalized derivatives, RaAc (**3**) and RaIAE (**4a**), and di-functionalized RaDiIAE (**4b**) were selectively isolated, achieving high purity (100% by NMR) but moderate yields (35%, 56%, 33%).

#### Ra-PEG conjugates

In the second step, the mono-functionalized Ra derivatives ((**3**) and (**4a**)) were coupled to dPEG-2SH (**1**) or dPEG-4SH (**2**) to form drug-carrier compounds. The conjugation reactions were performed at a stoichiometric ratio of Ac or IAE groups to SH groups, theoretically leading to a single Ra molecule being attached per PEG carrier molecule, and confirmed by UV measurement of Ra in the compounds.

#### Hydrogel formation

The PEGylated Ra derivatives, RaAc-dPEG-SH (**5**), RaIAE-dPEG-SH (**6**), RaAc-dPEG-3SH (**7**), and RaIAE-dPEG-3SH (**8**), were subsequently employed in hydrogel formation. Crosslinking was achieved using hydrolytically degradable 2dPEG-2SH (**1**) or 10dPEG-4SH (**2**), in combination with multi-arm PEG macromers 20PEG-8VS (**9**) or 20PEG-8Ac (**10**), yielding two distinct hydrogel assemblies (A and B). Additionally, a third gel assembly (C) was formed through copolymerization of RaDiIAE (**4b**) with crosslinker (**2**). These gels exhibited distinct structural and functional properties, enabling a comprehensive evaluation of swelling and controlled drug release.

### Swelling

#### Gel assembly A

**Figure 4.**
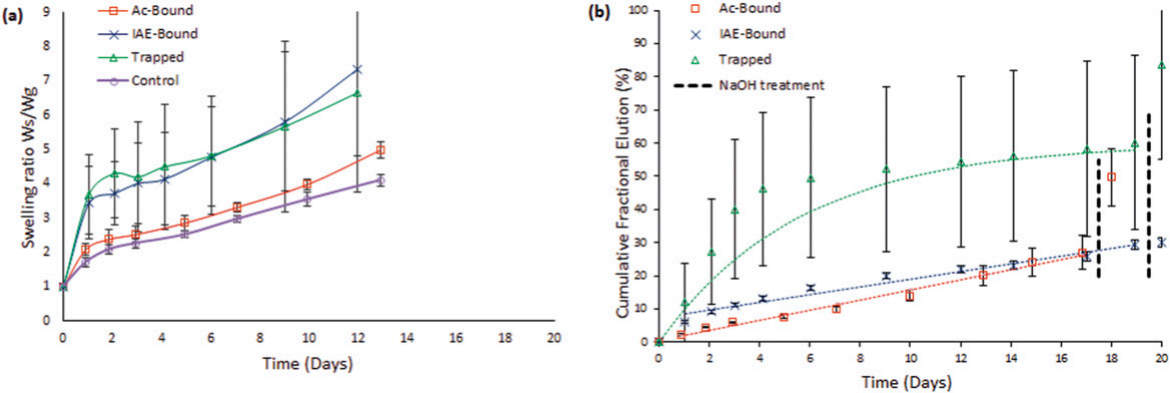
Gravimetric swelling ratios (a) and Cumulative rapamycin elution (b) for Assembly A gels (RaAc or RaIAE, PEGylated with 2dPEG-2SH, and co-polymerized with 2dPEG-2SH and 20PEG-8VS).

Assembly A (Figure 4(a)) gels reached swelling equilibrium by day 2. At this point, the Control and Ac-Bound gel variants exhibited a swelling degree of approximately 2 (swollen weight over initial gel weight), while the Trapped and IAE-Bound variants reached a swelling degree close to 4. Swelling then progressed gradually until manual handling was no longer possible after day 12 (Trapped and IAE-Bound gels) and day 13 (Control and Ac-Bound gels).

#### Gel assembly B

**Figure 5.**
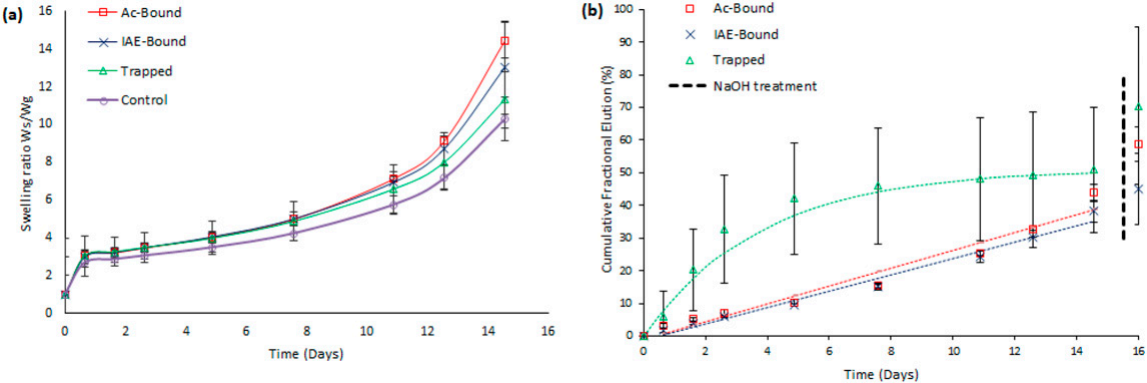
Gravimetric swelling ratios (a) and cumulative rapamycin elution (b) for Assembly B gels (RaAc or RaIAE, PEGylated with 10dPEG-4SH, and co-polymerized with 10dPEG-4SH and 20PEG-8Ac).

For Assembly B gels (Figure 5(a)), all four variants exhibited similar initial swelling behavior, reaching a distinct plateau after day 1 with a swelling ratio of approximately 3. From that point onwards, swelling increased moderately until day 8, reaching swelling ratios close to 5. Thereafter, the swelling ratio increased rapidly, reaching values between 10 and 15 by day 15, at which point the gels had become too fragile to allow reliable weight measurements.

#### Gel assembly C

**Figure 6.**
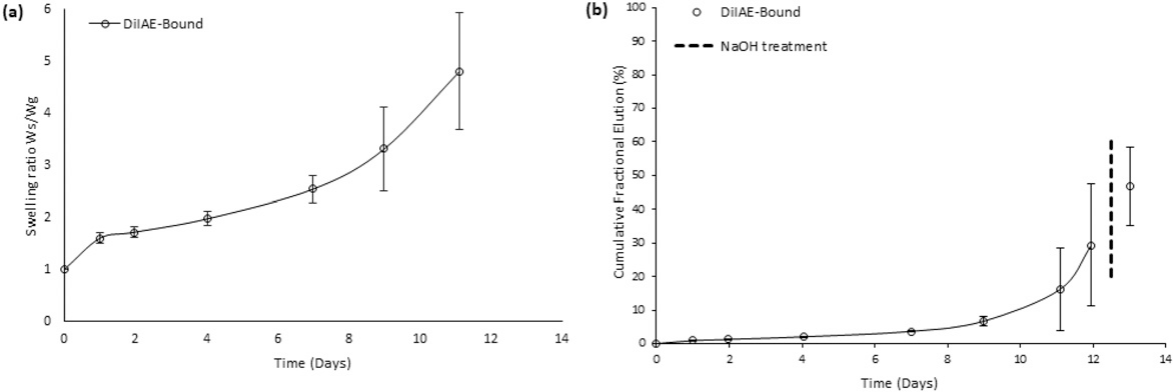
Gravimetric swelling ratios (a) and cumulative rapamycin elution (b) for Assembly C gels (non-PEGylated RaDiIAE co-polymerized with 10dPEG-4SH).

Assembly C gels (Figure 6(a)) exhibited a triphasic swelling behavior. After the initial phase, a distinct plateau was reached after the first day with a swelling ratio of approximately 1.6. This was followed by a second phase of moderate swelling, during which the ratio gradually increased to about 2.5 by day 7. In the third phase, rapid gel degradation led to a sharp rise in swelling, reaching a ratio of approximately 5 by day 11, at which point the gels could not be handled any longer.

### Rapamycin elution

#### Gel assembly A

Hydrolytic release of covalently incorporated Ra occurred over 17 days for the Ac-Bound gels and 19 days for the IAE-Bound gels (Figure 4(b)). Both release profiles followed zero-order kinetics (R^2^ = 0.99 and 0.97, respectively). The release rate was 11% faster from the IAE-Bound gels, which contain α-thioether ester bonds (k_0_ = 0.079 mg Ra/g gel/day), compared with the Ac-Bound gels containing β-thioether ester bonds (k_0_ = 0.071 mg Ra/g gel/day). These zero-order release rate constants differed significantly between the two linkers within each assembly (unpaired Student’s t-test, p < 0.05), confirming that the chemical linkage influenced the hydrolysis-driven release kinetics.

A rapid initial burst release occurred from Trapped formulations during the first 3 days leading to 67% of the total drug release being reached, while the release rate declined substantially thereafter. However, when the full release profile was fitted to a first-order model, the linearity of ln (fractional remaining release) vs time met the predefined criterion (R^2^ > 0.95) and thus the release is classified as first-order (Figure 4(b)). In contrast, the cumulative release from the ‘Bound’ formulations increased steadily throughout the 19-days study period; by day three, only 22% and 38% of the total release had been reached for the Ac-Bound and IAE-Bound gels, respectively.

The total cumulative drug release, post NaOH treatment, amounted to 50% of the initial loaded drug for Ac-Bound gels, 32% for IAE-Bound gels and 67% for Trapped gels.

#### Gel assembly B

Drug release followed zero-order kinetic profiles over a 15-day period for both Ac-Bound and IAE-Bound formulations (R^2^ = 0.95 and 0.98, respectively; Figure 5(b)). The release rate was 31% higher in the IAE-Bound gels (k_0_ = 0.17 mg Ra/g gel/day) compared to Ac-Bound gels (k_0_ = 0.13 mg Ra/g gel/day). These differences in k_0_ were statistically significant (unpaired t-test, p < 0.05).

Ac-Bound hydrogels released 50% of the total cumulative drug release after 10 days, while IAE-Bound gels reached the same level a day earlier.

The Trapped gel formulation followed first-order release kinetics (R^2^ = 0.98). After initial rapid release, 83% of the total cumulative release was reached by day 5 (compared to day 15), with no significant additional elution until the endpoint at day 15. Conversely, drug release from the ‘Bound’ formulations, steadily continued over the entire elution period. By day 5, only 23% and 15% were released by the Ac-Bound and IAE-Bound gels, respectively.

Ac-Bound gels released 59% of their initial drug content after NaOH treatment, while IAE-Bound gels released 48% and Trapped gels 71%.

#### Gel assembly C

During the synthesis of RaIAE (**4a**), a di-substituted byproduct ((RaDiIAE (**4b**)) was isolated and characterized. This derivative presents two reactive sites and may thus act as a crosslinker. Consequently, non-PEGylated RaDiIAE (**4b**) was incorporated into a gel using compound (**2**) as the multi-arm crosslinker.

Ra release from these gels (Figure 6(b)) exhibited zero-order kinetics for the first 7 days (R^2^ = 0.99, k_0_ = 0.17 mg Ra/g gel/day). Thereafter, the drug release rate rapidly increased and the elution trend showed a strong exponential burst character. The theoretically calculated drug content of the gels was 37 mg Ra per gram, of which 29% was released before NaOH addition, and a further 18% following forced degradation of the gels.

## Discussion

### Synthesis of gel precursors

In the synthesis of Ra derivatives, the choice of AcCl as an acrylation agent presented certain challenges. The use of two equivalents of AcCl favored mono-substitution, but resulted in low conversion efficiency. To improve conversion rates, the addition of more AcCl (up to 8 equivalents) was explored, which led to complete conversion, but also increased the formation of di- and tri-substituted products. Tai et al.^30^ steered away from direct acrylation to avoid such a mixture and opted for a tedious process involving the use of protective groups. We isolated mono-substituted Ra from the mixed product using simple column chromatography. The outcome of the acrylation reaction (in terms of substitution degree) was difficult to control, likely due to competition between TEA and AcCl, leading to formation of an insoluble yellow precipitate. This adverse reaction could be suppressed by adding AcCl dropwise from a dilute solution until complete Ra consumption could be confirmed by TLC. The need for excess AcCl may stem from its consumption in precipitate formation or the reaction atmosphere not being sufficiently dry, leading to AcCl hydrolysis.

During RaIAE synthesis, both mono- and di-substituted products were formed, but tri-substitution was not observed. The carbodiimide coupling reagent is less prone to nucleophilic attack than AcCl, rendering the sterically hindered hydroxyl group at position 10 of Ra unreactive towards carbodiimide-coupling. Literature supports this observation, indicating that the hydroxyl at position 40 is the most reactive, while position 10 is the least reactive.^38^

A key structural difference between the AcCl and IAA modifications lies in the molecular length of the introduced linker section between the drug and the PEG-carrier. The AcCl modification introduces a two-carbon spacer between the ester and thioether groups, whereas the use of IAA results in a single-carbon separation. The electron-withdrawing effect of the thioether group in closer proximity to the ester contributes to enhanced ester hydrolysis.^18^ This variation in linker length significantly influences the physicochemical properties and behavior of the final conjugates, as will be discussed later.

Conjugation of the mono-functionalized Ra derivatives ((**3**) and (**4a**)) with PEG was performed to enhance water solubility of the highly lipophilic drug (2.6 μg/ml). A series of Ra-PEG conjugates were synthesized, and 1.5 kDa was found to be the minimum PEG chain length required for solubilization of Ra. The PEGylated water-soluble drug-carrier compounds had a solubility of at least 180 mg/mL in phosphate-buffered saline (PBS).

A two-arm, 2 kDa PEG (2dPEG-2SH**,** (**1**)) was selected as the linker molecule for compounds (**5**) and (**6**), which was subsequently used in Assembly A gels (RaAc-SH and RaIAE-SH). However, a two-arm PEG molecule carried the risk of di-substitution, which could lead to insoluble conjugates lacking SH functional groups for gel incorporation. By contrast, using a four-arm 10 kDa PEG (10PEG-4SH (**2**)) allowed for multi-substitution while maintaining solubility and ensuring at least one SH group remained available for crosslinking. Consequently, compound (**2**) was chosen for Assembly B gels (RaAc-3SH and RaIAE-3SH). To counteract the increased gel stability associated with multi-arm carriers, a hydrolytically degradable crosslinker (20PEG-8Ac, (**10**)) was employed, rather than the stable 20PEG-8VS (**9**) crosslinker in Assembly A gels.

### Hydrogel swelling

Hydrogel swelling ratios were determined based on wet weight (W_s_/W_g_) rather than volume-based measurements. This approach was chosen for practical reasons: (i) Mass-based tracking allowed for prolonged swelling assessments, particularly when gels became too degraded for submersion in ethanol, a step required for volume measurements. (ii) The high water content of swollen gels (>90%) results in negligible differences between mass- and volume-based calculations. (iii) Regular weighing of the same gel samples throughout the experiment conserved materials and ensured consistent tracking of swelling trends.

Degradable PEG hydrogels often exhibit a typical characteristic triphasic swelling pattern.^39^ In the first phase, the gels exhibited rapid water uptake, continuing until swelling equilibrium was reached within 1-2 days. This initial plateau represents a thermodynamic balance between osmotic forces driving water uptake and the elastic retraction of the polymer network resisting expansion. The equilibrium state depends on the crosslinking density, hydrophilicity, and network homogeneity of each gel. During the second phase the swelling rate leveled off and remained nearly constant over several days as hydrolysis of cleavable linkages began to loosen the network. In the third and final phase, the swelling rate increased again as degradation progressed due to more aggressive hydrolytic breakdown of the polymer network, which led to structural weakening and facilitated further water absorption.

The duration and intensity of each phase is strongly influenced by gel composition, crosslinking chemistry, and network structure. These swelling profiles provide insight into the mechanical integrity and degradation pathways of the different formulations.

After reaching equilibrium swelling, Assembly A gels maintained stable swelling profiles throughout the degradation period (Figure 4(a)). No third-phase accelerated swelling was observed, even at late time points. This behaviour is attributed to the presence of VS-based crosslinking, which forms highly stable thioether bonds. These bonds resist hydrolysis, limiting network degradation and thereby restricting further swelling. Among Assembly A gels, the Ac-Bound variant swelled less than the IAE-Bound gels. This difference likely stems from the chemical structure of the linkers - the acrylate-based linker introduces two methylene groups between the ester bond and the polymer, offering greater steric protection compared to the shorter IAE linker with only one methylene group, resulting in slower degradation and reduced swelling.

Assembly B gels (Figure 5(a)) exhibited a third swelling phase with marked increase in swelling ratio during the final days. Using multi-arm acrylated PEG macromer (**10**), instead of multi-arm PEG-VS (**9**), in the RaAc/IAE-3SH assemblies doubled the number of cleavable ester groups per crosslink. Additionally, replacing crosslinker (**1**) with crosslinker (**2**) increased both the number of degradable crosslinking sites and the spacing between them. This resulted in a gel network that is more susceptible to hydrolysis and progressive degradation lead to the accelerated expansion of the gels. After day 11 extensive network breakdown caused more rapid increase in swelling ratio until the gels fell apart completely.

The two Ra-containing gel variants in Assembly B showed similar swelling behaviour up to day 11, suggesting that early swelling was largely governed by the degradation of acrylate-based linkages, rather than by differences in Ra-conjugate chemistry.

Assembly C gels (Figure 6(a)) underwent a sharp increase in swelling ratio in the final, third phase, likely due to rapid network collapse and loss of mechanical integrity around day 7. Incorporation of RaDiIAE (**4b**) as a crosslinker, which likely increases local hydrophobicity around crosslinking sites (compared to PEG-based crosslinkers), may reduce local water availability in the gel, thereby slowing hydrolytic degradation, as shown experimentally by Schoenmakers et al.^18^ However, the onset of hydrolytic degradation led to a sudden loss of integrity and accompanying uncontrolled water absorption and disintegration.

Across Assemblies A and B, the Control gels, lacking Ra, consistently exhibited lower swelling than their drug-loaded counterparts. This suggests that the presence of Ra, whether covalently bound or physically entrapped, interferes with network formation. Steric hindrance or consumption of crosslinking sites by the Ra conjugates in the gel matrix likely reduces crosslinking efficiency. Hydrophobic, trapped Ra may also disrupt gel network formation, resulting in looser networks with greater swelling capacity.

### Drug elution

Drug elution profiles were compared to well-established mathematical models. Zero- and first-order release kinetics were confirmed if data corresponded with the relevant rate equations. A coefficient of determination (R^2^) above 0.95 was considered a significant fit. If the majority of the drug was released within the first 2 days, the release profile was categorized as a burst release.

The Hixson-Crowell equation describes degradation of a gel as the primary drug release mechanism.^40^ Differentiation between diffusion- and swelling-driven release was achieved using the Korsmeyer-Peppas equation.^40,41^

All release experiments in this study were performed in PBS, which is widely used for initial characterization of drug-loaded hydrogels. Although biological media may influence hydrogel degradation, previous studies on PEG-based networks have shown minimal differences in drug release profiles between PBS and serum-containing media,^42^ as well as between PBS and simulated body fluid or other non-serum physiological buffers.^43,44^ Additional work on PEG-based drug carriers has similarly reported that hydrolysis of ester linkages, rather than enzymatic processes, governs the release mechanism under physiological conditions,^21,22^ and that the rate of ester hydrolysis remains comparable across buffered aqueous environments.^42–44^ Enzymatic effects are expected to be minor, since PEG lacks natural proteolytic or lipolytic targets and exhibits minimal susceptibility to enzymatic degradation.^19^

Moreover, the gels are intended to be injected intramuscularly, not directly into the bloodstream. Although hydrophobic drugs like Ra does bind to proteins, and may lead to lower free drug concentration, protein concentrations in interstitial fluid are substantially lower than in serum, making significant protein-mediated alterations in release unlikely.^44^ In more complex biological environments, slight deviations may occur, for example, early acceleration due to protein-mediated wetting or later reductions due to surface fouling,^42^ but such effects are typically minor relative to the dominant hydrolytic mechanism. Importantly, the zero-order hydrolysis-driven release kinetics observed here are consistent with previous reports on ester-linked PEG hydrogels.^42^ Together, these data support the use of PBS as a reliable medium for preliminary comparison of the gel architectures studied, while future work in serum or simulated physiological media may provide additional confirmation of translational robustness.

Incomplete drug release from hydrogels is commonly reported in the literature, yet no conclusive explanation has been established for systems with either covalently bound^42,44^ or physically entrapped^45,46^ drugs. In the present study, the apparently incomplete recovery of Ra (32–59% of the theoretical loading after NaOH treatment) is not attributed to any loss of UV absorbance upon PEG conjugation, as Ra–PEG conjugates retain a clear and quantifiable signal at 279 nm. Instead, the most likely explanation is an overestimation of the theoretical drug loading, since the preparation of highly concentrated Ra–PEG stock solutions did not account for the solid-volume contribution, thereby inflating the calculated Ra content. A second contributing factor may be partial interference from PEG dissolution during the NaOH treatment, which can reduce apparent UV-detectable Ra concentrations.^42^ In addition, direct UV spectroscopy is less sensitive than chromatographic methods at low (µg/mL) Ra concentrations and may be affected by gel fragments or degradation products in the solution; therefore, HPLC or LC-MS quantification would be expected to provide higher analytical sensitivity and a more accurate estimate of residual Ra.^47^ Finally, Ra is susceptible to base-catalyzed degradation, and partial decomposition during the forced-degradation step may further reduce the fraction of detectable Ra. We cannot fully exclude that a fraction of Ra remains covalently bound in sterically shielded regions of the network even after NaOH treatment, but we consider this a minor contributor relative to the analytical factors outlined above.

Both Ac-Bound and IAE-Bound gels of Assembly A exhibited zero-order release kinetics (R^2^ = 0.986 and R^2^ = 0.966, Figure 4(b)). Comparing the linear phases, the drug release rate was approximately 11% faster from IAE-Bound gels than in Ac-Bound gels (rate coefficient k_0_ = 0.079 mg Ra/g gel/day vs k_0_ = 0.071 mg Ra/g gel/day). This variation can be attributed to differences in linker structure between RaAc-dPEG-SH (**5**) and RaIAE-dPEG-SH (**6**). The linker in compound (**5**) has a two-carbon spacing between the thioether and ester groups, whereas compound (**6**) has only one. Even this small structural difference influences hydrolysis rates, as previously observed by Schoenmakers et al., reporting that a single-carbon reduction significantly accelerated drug release.^18^

Entrapped Ra was released faster than covalently bound Ra, with a release and swelling profile consistent with that previously reported for degradable PEG hydrogels.^39^ Although Trapped gels followed first-order kinetics (R^2^ = 0.950), neither the Hixson-Crowell nor Korsmeyer-Peppas equations provided a conclusive release mechanism. Drug release continued after swelling equilibrium was reached, then plateaued while swelling ratio increased, and was not further affected by the increased degradation rate starting at day 11. This suggests that diffusion was the primary release mechanism, rather than swelling or degradation.

For Assembly B gels elution rates also differed between Ac-Bound and IAE-Bound gels (Figure 5(b)), with the latter releasing drug approximately 31% faster (release coefficient k_0_ = 0.17 mg Ra/g gel/day vs k_0_ = 0.13 mg Ra/g gel/day). This suggests that both gel architecture and the linker structure (number of methylene groups separating the thioether and ester groups) influence hydrolysis rates.

Padsalgikar et al.^48^ described how the rate of hydrolysis is influenced not only by susceptible chemical bonds, but also by the internal water concentration within the material. The high surface area-to-volume ratio of a porous material can contribute to accelerated hydrolysis. Mesh size calculations indicated that transitioning from 20PEG-8VS (**9**) to 20PEG-8Ac (**10**) multi-arm PEG significantly increased gel porosity (unpublished results). Thus, it is hypothesized that incorporating crosslinker (**10**) led to a more porous gel structure, also contributing to the observed faster hydrolysis rate in Assembly B hydrogels. Although this may not significantly affect initial swelling, increased cleavage of drug molecules contributes to faster release rates.

For Assembly B Trapped formulations, elution data fit first-order kinetics (R^2^ = 0.978) but did not align with the Hixson-Crowell or Korsmeyer-Peppas models, precluding direct assignment of release mechanism to degradation, diffusion, or swelling. However, considering elution and swelling profiles (Figure 5(a) and (b)), swelling equilibrium was reached on the first day, whereas constant drug release continued until day three. Moreover, drug release rate declined dramatically while gel degradation continued (days 3–15), indicating that diffusion was the dominant release mechanism. In contrast, ‘Bound’ formulations followed hydrolysis-driven release kinetics.

Release profiles from Assembly C gels followed a biphasic pattern (Figure 6(b)), consisting of an initial zero-order release phase followed by a final burst phase. A similar biphasic release pattern, predicted through mathematical modelling, was experimentally validated in degradable conjugate addition gels containing covalently incorporated fluorophores.^20^ The late-stage accelerated release was attributed to gel dissolution near the endpoint. As shown in Figure 6, the sharp increase in Ra release, starting after approximately 7 days, coincided with an increase in swelling rate, suggesting that the onset of hydrogel degradation contributed to the final burst phase. However, in comparison to findings by DuBose et al.,^20^ the biphasic nature of release was more pronounced in the present study. This discrepancy is likely due to intrinsic differences in hydrogel composition, particularly the use of a degradable PEG thiol. Supporting this hypothesis, DuBose et al. demonstrated that increasing crosslink density reduced biphasic behavior, favoring prolonged zero-order release.

Apart from the non-linear drug release profile, another limitation of the RaDiIAE hydrogel assembly is that non-PEGylated Ra is not water-soluble, requiring gelation to occur in a mixed organic-aqueous solvent system. As a result, these hydrogels are not suitable for in situ gelation, limiting their application in injectable formulations.

These findings highlight how swelling behaviour and drug release is determined not only by macromer design and degradation chemistry, but also by the physicochemical properties of the drug and its mode of incorporation. Monitoring swelling offers valuable insight into gel network evolution and functional lifetime, particularly in relation to structural stability and onset of degradation. Release profiles give an indication of the therapeutic suitability of the gel as drug carrier vehicle.

Results presented confirm that controlled zero-order release of rapamycin from a hydrogel can deliver constant levels of drug at the target site over a period of time. Drug architecture can be tailored to supply the required therapeutic levels over the desired time period. Hydrogels thus provide a highly tunable scaffold for achieving desired local drug administration, thereby reducing dosing frequency, improving local bioavailability, minimizing systemic toxicity and fluctuating drug levels. Such sustained delivery has been shown to inhibit smooth muscle cell proliferation and prevent neointimal hyperplasia more effectively than free rapamycin, as demonstrated in nanoparticle-hydrogel systems applied periadventitially in vascular injury models.^49^

## Conclusions

This study developed three distinct hydrogel assemblies by employing different crosslinkers, multi-arm drug carriers, and crosslinking strategies, enabling systematic investigation of Ra release from degradable PEG networks. In formulations where Ra was covalently incorporated (Ac-Bound and IAE-Bound), drug release followed zero-order kinetics, and the influence of linker structure was evident, with IAE-Bound gels exhibiting faster hydrolysis and release rates than their Ac-Bound counterparts. Gel architecture further modulated these kinetics, demonstrating that network design can extend or amplify the effects of the chemical linker. Zero-order release could be maintained for 7–19 days with k_0_ values tunable between approximately 0.07 and 0.17 mg Ra/g gel/day, depending on linker structure and gel architecture. In contrast, drug release from Trapped formulations was governed primarily by diffusion. Collectively, these findings clarify the mechanistic origins of both sustained and burst-like release behaviours and show that linker chemistry and gel architecture can be engineered to tune Ra release from rapid initial elution to prolonged zero-order kinetics.

Despite these promising results, limitations such as discrepancies in detected drug amounts and instances of incomplete release highlight the need for further refinement of experimental methodology and deeper understanding of drug–gel interactions. Future work will include investigation of biologically relevant elution mediums, more advanced measurement techniques, and the study will be expanded to include evaluation of cellular and in vivo responses to drug release.

Nonetheless, the tunable, zero-order local delivery over 1–3 weeks achieved in this study, directly aligns with clinical requirements in applications such as post-myocardial infarction intramyocardial therapy and vascular interventions, where maintaining stable rapamycin levels are desirable, while minimizing systemic exposure. The tunability provided by linker structure and gel design offers a modular platform for improving outcomes in such applications. Together, these insights lay a solid foundation for future advancements in hydrogel-based controlled drug delivery systems.

## Supplemental Material

Supplemental Material - Engineered zero-order drug release from degradable PEG hydrogels – A rapamycin case study Supplemental Material for Engineered zero-order drug release from degradable PEG hydrogels – A rapamycin case study by Lage Ahrenstedt, Anel Oosthuysen, Peter Zilla, Jaco Theron, Deon Bezuidenhout in Journal of Biomaterials Applications.

## Data Availability

The datasets generated and analyzed during this study are available from the corresponding author on reasonable request. *
