# Supplementary material for: Engineered zero-order drug release from degradable PEG hydrogels – A rapamycin case study
Source: J Biomater Appl. 2026 Jan 6;41(1):29–42. doi: 10.1177/08853282251410673 (PMC13234310; doi:10.1177/08853282251410673)
Supplement: Supplemental Material - Engineered zero-order drug release from degradable PEG hydrogels – A rapamycin case study [file sj-pdf-1-jba-10.1177_08853282251410673.pdf]

Supplementary Information

S1) <u>Synthesis of 2dPEG-2SH</u> (**1**; d = hydrolytically degradable; SH = thiol group):
2 kDa linear 2-armed PEG (2PEG-2OH; 10 mmol OH), 3-mercaptopropionic acid (49 mmol) and para-toluene sulphonic acid (pTSA; 0.50 mmol) were dissolved in toluene (150 ml). The reaction mixture was heated (140° C) and refluxed in a Dean-Stark apparatus overnight. The solution was cooled on ice and the volume reduced prior to precipitation in cold diethyl ether. The crude product was re-precipitated from dichloromethane (DCM; 20 ml) into cold diethyl ether (200 ml) and the collected product was vacuum dried (9.9 g, 90% yield, 99% purity by nuclear magnetic resonance; NMR).

^{1}H NMR (300 MHz, $CDCl_3$): δ= **1.62** (t, J= 8.1 Hz, 2H; -S*H*), **2.59-2.64** (m, 4H; -OCOC$H_2$$CH_2$SH), **2.67-2.75** (m, 4H; -OCOCH_2CH_2SH), **3.32-3.82** (m; PEG) and **4.20** (t, J= 4.7 Hz, 4H; PEG-CH_2CH_2OCO-) ppm. ^{13}C NMR (75.5 MHz, $CDCl_3$): δ= **19.50** (-CH_2*C*H_2SH), **38.21** (-*C*$H_2$$CH_2$SH), 63.53, 68.84, 70.35 and **171.26** (-*C*OO-) parts per million (ppm).

1

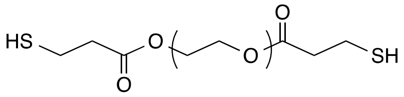

S2) <u>Synthesis of 10dPEG-4SH (2):</u>
10 kDa star-shaped 4-armed PEG (10PEG-4OH; 3.8 mmol OH), 3-mercaptopropionic acid (40 mmol) and pTSA (0.40 mmol) were dissolved in toluene. The reaction mixture was heated (140° C) and refluxed in a Dean-Stark apparatus overnight. The solution was cooled on ice and the volume reduced prior to precipitation in cold diethyl ether. The crude product was re-precipitated from DCM into cold diethyl ether and the collected product was vacuum dried (8.7 g, 88% yield, 99% purity by NMR).

^{1}H NMR (300 MHz, $CDCl_3$): δ= **1.64** (t, J= 8.4 Hz, 4H; -S*H*), **2.61-2.67** (m, 8H; -OCOC$H_2$$CH_2$SH), **2.69-2.78** (m, 8H; -OCOCH_2CH_2SH), **3.35-3-85** (m; PEG) and **4.23** (t, J= 4.7 Hz, 8H; PEG-CH_2CH_2OCO-) ppm.

2

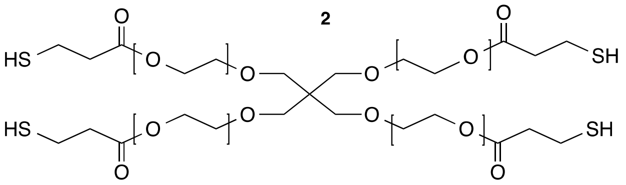

S3) <u>Synthesis of 20PEG-8VS</u> (**9**; VS = vinyl sulphone group):
20PEG-8OH (8.0 mmol OH) was dissolved in DCM (200 ml) and dried over molecular sieves (4 Ångström) overnight. The molecular sieves were removed by filtration and NaH (65 % in oil; 41 mmol) was added in portions followed by addition of DCM (300 ml). The solution was then purged with argon gas for five minutes by bubbling. Divinyl sulphone (398 mmol) was added in one portion, the solution was bubbled with argon (30 min) and left to react for two days in darkness. Addition of glacial acetic acid (44 mmol) quenched the remaining NaH and protonated the product. The volume was reduced to ~100 ml by rotary evaporation prior to precipitation in cold diethyl ether (1L). The product was collected on a filter and remaining solvent removed by vacuum. The precipitation was repeated twice to obtain a slightly off white powder (6.8 g, 32% yield, ~100% purity by NMR).

^{1}H NMR (300 MHz, $CDCl_3$): δ= **3.25** (t, J= 5.7 Hz, 16H; -PEGOCH$_2$C*H*$_2$VS), **3.58-3.72** (m; PEG), **3.89** (t, J= 5.7 Hz, 16H; -PEGOC*H*$_2$CH$_2$VS), **6.07** (d, J= 9.9 Hz, 8H; VS$_{Cis}$), **6.38** (d, J= 16.7 Hz, 8H; VS$_{Trans}$) and **6.80** (dd, J= 9.9 and 16.7 Hz, 8H; VS$_{gem}$) ppm.

9

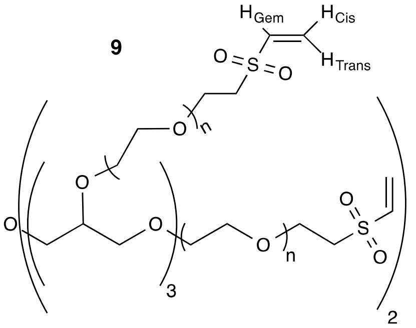

S4) <u>Synthesis of 20PEG-8Ac</u> (**10**; Ac = acrylate group):

20 kDa comb-shaped 8-armed PEG (20PEG-8OH; 4.0 mmol OH) was dissolved in toluene (500 ml) and dried by azeotropic distillation. The residue (~400 ml) was cooled on ice and diluted with DCM (200 ml). Freshly distilled triethylamine (10 ml, 10 mmol, out of a stock solution of 7 ml in 50 ml DCM) was added. Acryloyl chloride (10 ml of a stock solution of 4 ml in 50 ml DCM, 10 mmol) was added in portions (2 ml every 10 min) over a 50 minutes period. The reaction was left with stirring overnight. The reaction mixture was filtered to remove insolubilities and the volume reduced (to ~60 ml) prior to precipitation in ice-cold diethyl ether (600 ml). The precipitation was repeated twice from DCM (60 ml) into ice-cold diethyl ether (600 ml). The product was further purified by dialysis (molecular mass cutoff =1 kDa) and freeze-dried to obtain a slightly off white powder (6.9 g, 68% yield, 94% purity by NMR).

^{1}H NMR (300 MHz, $CDCl_3$): δ= **3.43-3.80** (m; PEG), **4.29** (t, J= 5.5 Hz, 16H; -PEGOCH$_2$C*H*$_2$Ac), **5.81** (dd, J= 10.4 and 1.3 Hz, 8H; Ac$_{Cis}$), **6.13** (dd, J= 17.4 and 10.5 Hz, 8H; Ac$_{Gem}$) and **6.40** (dd, J= 17.4 and 1.2 Hz, 8H; Ac$_{Trans}$) parts per million (ppm).

10

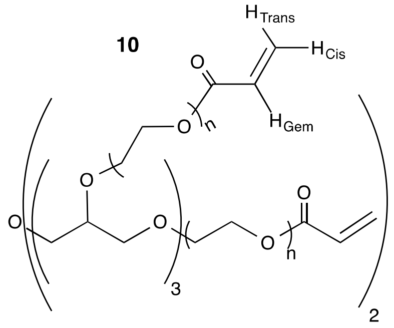

S5) <u>HPLC and ESI LC-MS characterization of compounds 3, 4a and 4b</u>

<u>HPLC retention times</u>

Ra reference 37.64 min, compound **3** 40.95 min, compound **4a** 41.07 min and compound **4b** 42.63 min.

<u>ESI LC-MS characterization</u>

Ra: (assigned) *m/z*= 814.5 (Ra fragment 1), 832.6 (Ra fragment 1 + H_2O), 846.6 (Ra fragment 2), 864.6 (Ra fragment 2 + H_2O), 882.6 (Ra fragment 2 + $2H_2O$), 931.6 (Ra + H_2O), 936.6 (Ra + Na^+), 952.6 (Ra + K^+) and 994.6 (Ra-adduct).
compound 3: (calculated, assigned): *m/z*= 886.6 (886.6, Ra-Ac fragment 1 + H_2O), 900.6 (900.6, Ra-Ac fragment 2), 918.6 (918.6, Ra-Ac fragment 2 + H_2O), 936.6 (936.6, Ra-Ac fragment 2 + $2H_2O$), 990.6 (990.6, Ra-Ac + Na^+) and 1006.6 (1006.6, Ra-Ac + K^+).
compound 4a: (calculated, assigned): *m/z*= 1000.5 (1000.5, RaIAE fragment 1 + H_2O), 1014.5 (1014.5, RaIAE fragment 2), 1032.5 (1032.5, RaIAE fragment 2 + H_2O), 1050.5 (1050.5, RaIAE fragment 2 + $2H_2O$), 1099.6 (1099.5, RaIAE + H_2O), 1104.5 (1104.5, RaIAE + Na^+), 1120.5 (1120.5, RaIAE + K^+) and 1162.5 (1162.5, Ra-adduct-IAE).
compound 4b: (calculated, assigned): m/z= 1014.5 (1014.5, Ra-diIAE fragment 2), 1182.4 (1182.4, Ra-diIAE fragment 2), 1200.4 (1200.4, Ra-diIAE fragment 2 + H_2O), 1218.4 (1218.4, Ra-diIAE fragment 2 + $2H_2O$), 1267.5 (1267.4, Ra-diIAE + H_2O), 1272.4 (1272.4, Ra-diIAE + Na^+), 1288.4 (1288.4, Ra-diIAE + K^+) and 1330.4 (1330.4, Ra-adduct-diIAE).
